# Facile Preparation of EVOH-Based Amphoteric Ion Exchange Membrane Using Radiation Grafting Technique: A Preliminary Investigation on Its Application for Vanadium Redox Flow Battery

**DOI:** 10.3390/polym11050843

**Published:** 2019-05-10

**Authors:** Kangjun Xie, Zhen Dong, Yicheng Wang, Wei Qi, Maolin Zhai, Long Zhao

**Affiliations:** 1 State Key Laboratory of Advanced Electromagnetic Engineering and Technology, School of Electrical and Electronic Engineering, Huazhong University of Science and Technology, Wuhan 430074, China; xiekangjun@hust.edu.cn (K.X.); zhendong@hust.edu.cn (Z.D.); qiwei@hust.edu.cn (W.Q.); 2 School of Chemistry and Chemical Engineering, Huazhong University of Science and Technology, Wuhan 430074, China; 3 Beijing National Laboratory for Molecular Sciences, Radiochemistry and Radiation Chemistry Key Laboratory of Fundamental Science, the Key Laboratory of Polymer Chemistry and Physics of the Ministry of Education, College of Chemistry and Molecular Engineering, Peking University, Beijing 100871, China; wang.yc@pku.edu.cn (Y.W.); mlzhai@pku.edu.cn (M.Z.)

**Keywords:** radiation grafting, ion exchange membrane, EVOH, VRFB

## Abstract

A novel amphoteric ion exchange membrane (AIEM) was successfully prepared by one-step radiation grafting of sodium styrene sulfonate (SSS) and dimethylaminoethyl methacrylate (DMAEMA) onto ethylene-vinylalcohol copolymer (EVOH) powder and sequent transferring into film by casting method. Fourier transform infrared spectrometry (FT-IR), thermal gravimetric analyzer (TGA) and elemental analysis testified SSS and DMAEMA were successfully grafted onto EVOH. The ion exchange capacity, water uptake and proton conductivity of the resulting AIEM increased with grafting yield (GY). At the GY of 40.9%, the permeability of vanadium ions of AIEM was 3.98 × 10^−7^ cm^2^ min^−1^, which was better than Nafion117 membrane. Furthermore, the cost of this AIEM is much lower than that of Nafion117 membrane. This work provided a low cost and simple method for fabrication of the ion exchange membrane for vanadium redox flow battery (VRFB). Meanwhile, it also provided a new direction for the application of EVOH.

## 1. Introduction

Recently, as a promising large-scale form of energy storage, the vanadium redox flow battery (VRFB) has attracted wide attention due to its high energy efficiency, long cycle life, high safety, low cost and environmentally friendly nature [1]. The VRFB operates by utilizing two redox reactions of V (II)/V (III) and V (IV)/V (V) couples in the negative and positive electrolytes separated by the ion exchange membrane [2]. The function of the ion exchange membrane is to allow transport of ions to complete the circuit and prevent the mixing of the negative and positive electrolytes [3]. As a key component of VRFB, a desirable ion exchange membrane should have high proton conductivity, good chemical stability, low vanadium ion permeability and low cost [4]. For example, as a most widely used commercial membrane, Nafion membrane has high proton conductivity and good chemical stability [5]. However, the high vanadium ion permeability of the Nafion membrane causes self-discharge of VRFB and results in decreasing of energy efficiency of VRFB [6]. Hence, there have been extensive research activities towards the modification of Nafion-based membrane to reduce its vanadium ion permeability. For instance, SiO_2_ [7], polypyrrole [8], fluorocarbon surfactant [9], sulfonated graphene oxide [10], polybenzimidazole [11], phosphotungstic acid immobilized nanofibers [12] and sulfonated copper phthalocyanine [13] were used to modify Nafion membranes and the modified Nafion membranes show lower vanadium ion permeability than pristine Nafion membranes. However, the high price of Nafion membranes (500–700 dollar m^−2^) hinders their applications [14]. Accordingly, developing a novel ion exchange membrane to provide a balance between the performance and cost is of great importance for VRFB industrialization.

The radiation induced grafting technique is an efficient method to prepare ion exchange membrane because no catalysts or additives are required to initiate the reaction and grafting reaction can be easily adjusted by varying the radiation condition [15]. For example, polytetrafluoroethylene films [16] and polyvinylidene fluoride films [17] have been used to prepare ion exchange membrane by radiation grafting of styrene. These ion exchange membranes containing sulfonic groups show high proton conductivity for application in VRFB. However, these methods are inconvenient because fluorinated films should be grafted with styrene and further sulfonated with chlorosulfonic acid. A new study shows that sodium styrene sulfonate (SSS) can be grafted onto PVDF film by pre-irradiation method using dimethylformamide (DMF), and dilute sulfuric acid as the solvent [18]. It is regrettable that this method is not applicable to PVDF powder due to the solubility of PVDF powder in DMF. Moreover, the distribution of grafted chains across the membrane based film grafting is inhomogeneous, which may restrict the large-scale production of the ion exchange membrane [19]. Therefore, a new type ion exchange membrane with a better preparation method should be further investigated.

**Scheme 1:**
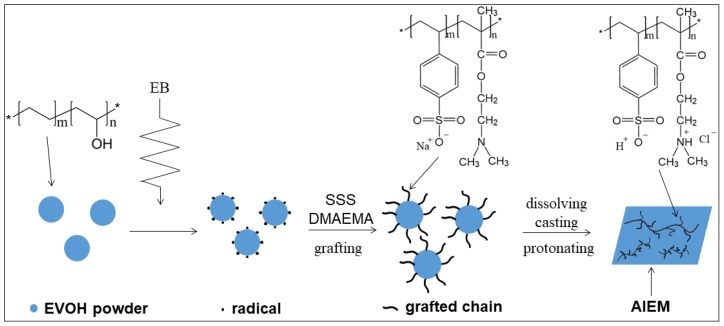
Synthesis route of AIEM.

Ethylene-vinylalcohol copolymer (EVOH) is a widely used polymer with good mechanical strength, high machinability and low cost [20]. At present, EVOH is mainly used for gas barrier and food packaging [21,22,23]. Many researchers are trying to apply EVOH to other fields such as polymer electrolyte [24], proton conductive membrane [25] and absorbent resin [26]. However, the application of EVOH for VRFB has not been reported. In this work, we report the first successful preparation of EVOH-based amphoteric ion exchange membrane (AIEM) by radiation grafting of SSS and dimethylaminoethyl methacrylate (DMAEMA) onto EVOH powder in an aqueous solution, and followed by dissolving, casting and protonation (Scheme 1). The characterizations of AIEM were investigated in detail using Nafion117 membrane for comparison. It is noteworthy that the cost of AIEM is about 50 dollar m^−2^ which is far below Nafion117 membrane. It is expected that this work will provide a simple method for preparation of AIEM with good performance for VRFB. Meanwhile, this work expands the application of EVOH.

## 2. Materials and Methods

### 2.1. Materials

E105B of EVOH was purchased from Kuraray Co., Ltd. (Tokyo, Japan), and then was grinded to the diameter about 100 um. E105B of EVOH shows excellent stability and machinability with an ethylene content of 44%. SSS, DMAEMA and dimethyl sulfoxide (DMSO) were purchased from Aladdin Chemical Co., Ltd. (Shanghai, China), vanadyl sulfate (VOSO_4_) was supplied by Aike Chemical Co., Ltd. (Chengdu, China). Nafion117 membrane was purchased from Du Pont (Wilmington, DE, USA). All chemicals were used as received without further purification.

### 2.2. Preparation of AIEM

The EVOH powder was flattened in polyethylene bag and vacuum sealed, and then it was irradiated with an electron beam accelerator (Wasik Associates INC, Boston, MA, USA) at the dose rate of 30 kGy/pass under dry ice cooling. Thereafter, the irradiated sample was placed in the deoxygenated mixed solution of SSS and DMAEMA. The grafting reaction was carried out at 70 °C for 2 h with continuous stirring and bubbling with nitrogen. The homopolymer and unreacted monomers were removed by washing with deionized water and dried at 60 °C to obtain a graft powder. The grafting yield (*GY*) was calculated using Equation (1),

(1) $$GY=\frac{W_{g}-W_{0}}{W_{0}}\times 100\%$$

where *W*_0_ and *W_g_* were the weight of EVOH powders before and after grafting, respectively.

The mole ratio of DMAEMA to SSS in the AIEM was determined according to Equation (2),

(2) $$\frac{n_{DMAEMA}}{n_{SSS}}=\frac{32\cdot P_{N}}{14\cdot P_{S}}$$

where *P_N_* and *P_S_* are the content of nitrogen and sulfur (wt %), respectively. 14 and 32 are atomic mass of nitrogen and sulfur, respectively.

The grafted powder was completely dissolved in DMSO to form a casting solution. Then the casting solution was applied to a clean glass plate and dried at 80 °C for 24 h to remove the solvent completely. After that, the grafted film was obtained on the glass plate. Finally, the grafted film was protonated for 6 h in 1 M HCl solution to obtain AIEM, and then kept in distilled water at room temperature prior to further use. Finally, the grafted film was protonated for 6 h in 1 M HCl solution to obtain AIEM, and then kept in distilled water at room temperature prior to further use.

### 2.3. Characterization

#### 2.3.1. Fourier Transform Infrared Spectrometry (FT-IR)

FT-IR spectra were measured using a spectrophotometer (Bruker Tensor 27, Karlsruhe, Germany) in attenuated total reflection mode, and the wavenumber range is 4000–650 cm^−1^ with a resolution of 2 cm^−1^.

#### 2.3.2. H-Nuclear Magnetic Resonance (^1^H-NMR)

^1^H-NMR spectroscopy of EVOH powder and grafted EVOH powder (GY = 15.2%) were tested by AscendTM 600MHz (Bruker, Karlsruhe, Germany). The solvent was dimethyl sulfoxide-d6. We selected the grafted EVOH powder with the grafting yield of 15.2% for testing because the viscosity of the solution of grafted EVOH powder with high grafting yield is too large to test.

#### 2.3.3. X-ray Diffraction (XRD)

X-ray diffraction patterns of original EVOH and AIEM were recorded using X-ray diffractometer (SmartLab-SE, Tokyo, Japan). The samples were measured at the diffraction angles (2θ) in the range of 10–80° using Cu Kα radiation.

#### 2.3.4. Thermogravimetric Analysis (TGA)

TGA was performed on a thermogravimetric analyzer (TA instrument mode 600) with the temperature range of room temperature to 800 °C and the heating rate of 10 °C min^−1^. The test was carried out in a nitrogen atmosphere.

#### 2.3.5. Element Analysis

The element composition of the grafted film and AIEM were investigated by Vario Micro cube (Elementar, Hanau, Germany).

#### 2.3.6. Surface Morphology

The surface morphology and elemental information of EVOH film and AIEM were investigated by scanning electron microscope equipped with an energy dispersive spectrometer (SEM-EDS) (Hitachi SU8000, Tokyo, Japan). The AIEM sample was sputtered with gold for 90 s under vacuum before the SEM-EDS measurements.

#### 2.3.7. Mechanical Properties

The mechanical properties of EVOH film, AIEM and Nafion117 membrane were carried out on an Electromechanical Universal Testing Machine (SEMtester 100, Milliren Technologies Inc., Newburypor, MA, USA). The dimensions of samples were 1 × 7 cm. The samples were stretched on 100 N mode.

#### 2.3.8. Water Uptake

In order to test the water uptake of AIEM, the dry membrane with ascertained weight was immersed into deionized water at room temperature for 24 h. After that, the wet membrane was taken out of the deionized water and wiped by filter paper. Then the weight of wet membrane was immediately accurately measured. The water uptake of AIEM was obtained by Equation (3),

(3) $$WU=\frac{W_{wet}-W_{dry}}{W_{dry}}\times 100\%$$

where *W_wet_* and *W_dry_* are weights of AIEM after and before swelling, respectively.

#### 2.3.9. Ion Exchange Capacity

The ion exchange capacity (IEC) of AIEM was determined by back titration. The sample membrane was dried at 40 °C for 24 h and weighted. Then, the dry membrane was immersed in a 0.05 M HCl solution overnight at room temperature. After that, the solution was back titrated with 0.5 M NaOH solution to be neutral. The IEC (mmol g^−1^) of the membrane was calculated by Equation (4),

(4) $$IEC=\frac{C_{HCl}\cdot V_{HCl}-C_{NaOH}\cdot V_{NaOH}}{W_{dry}}$$

where *C_HCl_* and *V_HCl_* are the concentration (mol L^−1^) and volume (mL) of the initial HCl solution, respectively. The *C_NaOH_* and *V_NaOH_* are the concentration (mol L^−1^) and volume (mL) of the consumed NaOH solution, respectively, and the *W_dry_* is the weight of the dried membrane.

#### 2.3.10. Proton Conductivity

The proton conductivity of the membrane was determined by electrochemical impedance spectroscopy (EIS) using a CHI660e electrochemical workstation (Shanghai Chenhua Instruments Co., China). The membrane was firstly immersed in 1 M H_2_SO_4_ solution at room temperature for 24 h. The membrane was clamped between two copper electrodes for testing the impedance spectroscopy. The frequency range and AC perturbation used in impedance spectroscopy are 1–100,000 Hz and 5 mV, respectively. The proton conductivity (*σ*) was calculated by Equation (5),

(5) $$\sigma =\frac{L}{RS}$$

where *R*, *L* and *S* are the resistance, thickness and area of the AIEM between the two copper electrodes, respectively.

#### 2.3.11. Permeability of V (IV) Ions Through the AIEM

**Figure 1:**
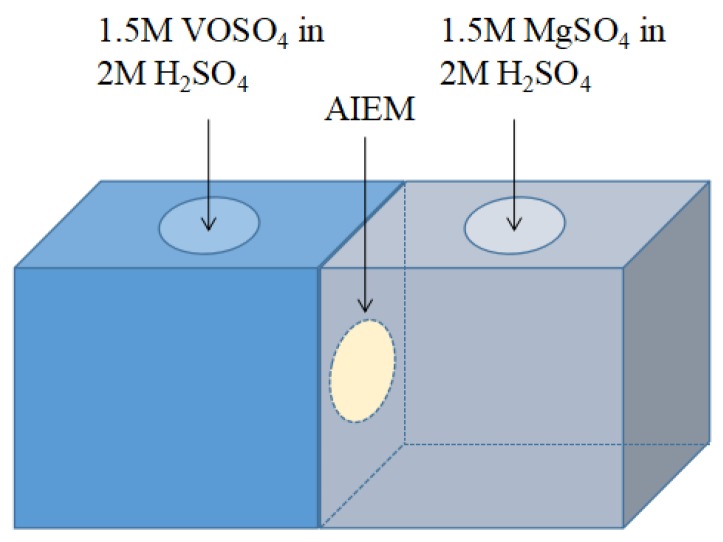
Illustration of the equipment for permeability of V (IV) ions test.

The permeability of V (IV) ions through the AIEM was investigated via the equipment as shown in Figure 1. As shown in Figure 1, a diffusion cell divided by a membrane was filled with 50 mL of 1.5 M VOSO_4_ in 2.0 M H_2_SO_4_ in the left half-cell and 50 mL of 1.5 M MgSO_4_ in 2.0 M H_2_SO_4_ in the right half-cell. The area of the AIEM exposed to the solution was 1.77 cm^2^. The concentration of V (IV) ions in the MgSO_4_ side was determined by for inductively coupled plasma atomic emission spectrometer (ICP-AES) (Leeman, Profile) at a regular time. The permeability of V (IV) ions through the Nafion117 membrane was also tested with same method.

#### 2.3.12. Open Circuit Voltage of the VRFB

The VRFB used was fabricated by sandwiching the membrane between two pieces of carbon felts (with a thickness of 3.0 mm) with an effective area of 9 cm^2^ on each side. The positive and negative electrolytes were 150 mL of 1.5 M V (IV) + 3.0 M H_2_SO_4_ and 75 mL of 1.5 M V (IV) + 3.0 M H_2_SO_4_, respectively. The electrolytes were cyclically pumped into the corresponding half-cell by peristaltic pump. The open circuit voltage of the VRFB was tested by 8 channels battery analyzer (BTS-8, Shenzhen Kejing Star Technology Co., Ltd., Shenzhen, China). The VRFB was first charged to 1.7 V at a current of 360 mA, and then the open circuit voltage was measured at room temperature.

## 3. Results

### 3.1. Radiation Grafting

**Figure 2:**
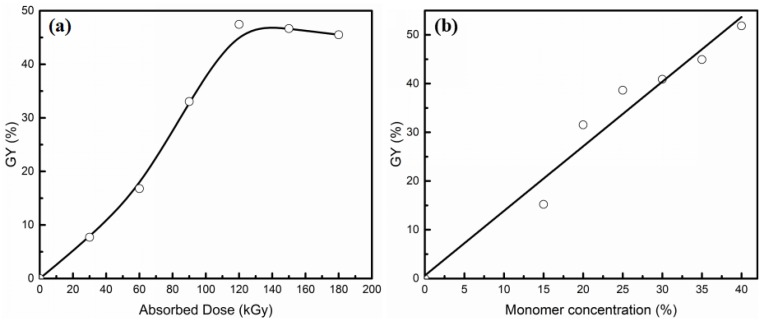
Effect of absorbed dose (**a**) and monomer concentration (**b**) on GY.

SSS and DMAEMA were grafted onto EVOH powder using one step electron-beam-induced pre-irradiation grafting technique. Due to the solvent of this grafting reaction being deionized water, the preparation process was simple and involved low pollution. The relationship between GY and absorbed dose is shown in Figure 2a. The total concentration of SSS and DMAEMA was 30 wt %, and the mole ratio of DMAEMA/SSS was 1/1. Firstly, the GY increases with the absorbed dose and reached a maximum of 47.5% at 120 kGy, and then GY decreases slightly with an increasing dose. This is due to radiation graft polymerization mainly being a free radical mechanism, and with an increase of the absorbed dose, more free radicals and reactive sites could be generated on the EVOH powder, which accelerated the grafting reaction. In the case of the free radicals generated on EVOH powder and the surface area of EVOH powder was limited, the GY of grafting reaction was restricted. The influence of total monomer concentration on GY is shown in Figure 2b. The absorbed dose was 120 kGy. It is found that GY increases with the increasing of monomer concentration. However, with the GY increasing, the solubility of grafted EVOH powder decreases in organic solvent. It is difficult to prepare the AIEM by grafted EVOH powder with too high GY. Therefore, providing a balance between the performance and GY is of great importance for AIEM.

### 3.2. Membrane Characterization

#### 3.2.1. FT-IR Analysis

**Figure 3:**
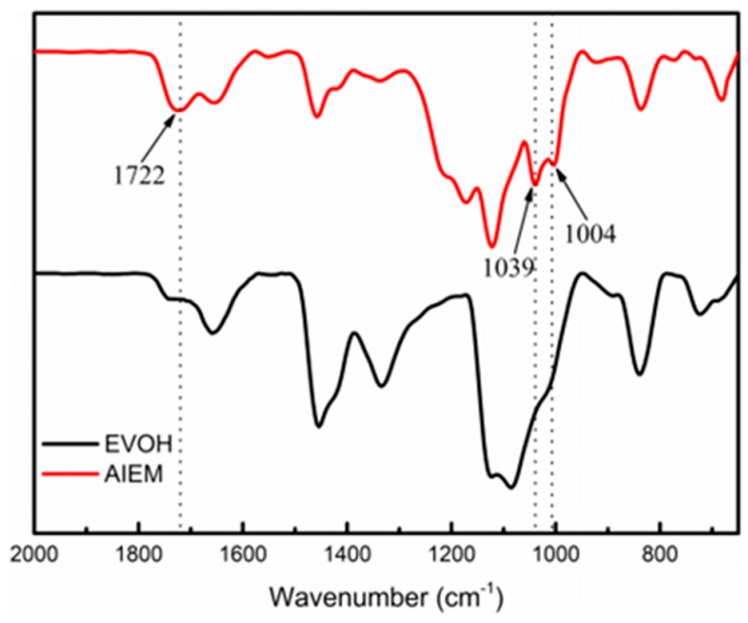
FT-IR spectra of original EVOH and AIEM (GY = 40.9%).

FT-IR spectra of the original EVOH and AIEM (GY = 40.9%) are shown in Figure 3. The grafting of SSS is verified by the characteristic absorption bands of sulfonic group at 1039 and 1004 cm^−1^. The absorption band at 1722 cm^−1^ can be assigned to the absorption of carbonyl group (C=O), which can confirm the successful grafting of DMAEMA. From FT-IR spectra, it can be confirmed that the AIEM was successfully prepared by radiation grafting method.

#### 3.2.2. H-Nuclear Magnetic Resonance (^1^H-NMR)

The ^1^H-NMR spectroscopy of EVOH powder and grafted EVOH powder (GY = 15.2%) are shown in Figure S1. The grafting of SSS is verified by the new peaks of 7.46 and 6.56 ppm, which are due to the hydrogen on the benzene ring. The new peaks of 4.30 and 2.62 ppm are assigned to (CH_2_–O–C=O) and (N–CH_2_) of DMAEMA, respectively. From the ^1^H-NMR spectroscopy it can be confirmed that the AIEM was successfully prepared by radiation grafting method.

#### 3.2.3. X-ray Diffraction

Figure S2 shows the XRD patterns of original EVOH and AIEM (see Supplementary Materials). The original EVOH and AIEM show only peak at 20.8 which is attributed to the (110) crystal plane of EVOH. The strength of the peak of AIEM is lower than original EVOH. It demonstrates that irradiation has slight damage to crystallinity of EVOH.

#### 3.2.4. TGA Measurement

**Figure 4:**
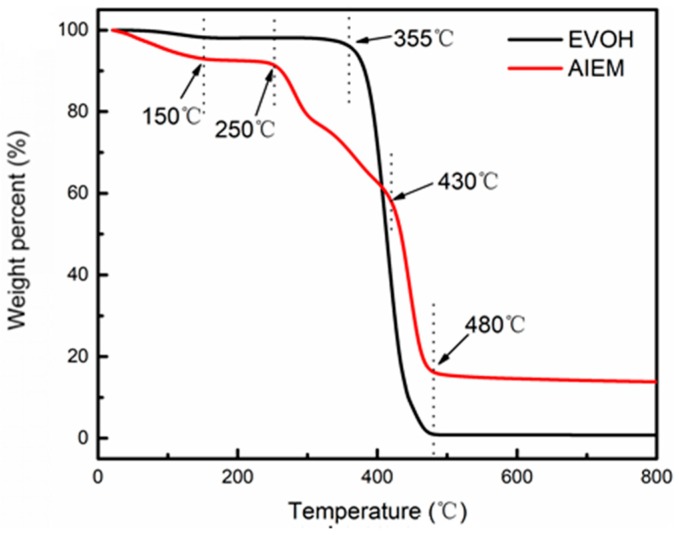
TGA curves of original EVOH and AIEM (GY = 40.9%).

The thermal stability of original EVOH powder and AIEM are shown in Figure 4. The thermal degradation behavior of EVOH powder involves only one-step process, and the weight loss stage are at 355–480 °C which is considered to be the degradation of EVOH backbone. For AIEM, there is obvious four-step degradation pattern in the curve of the AIEM. The first weight loss stage from temperature to 150 °C is corresponding to the weight loss of bound water in the AIEM. The second weight loss stage at 250~300 °C is due to the degradation of graft chain of DMAEMA. The third weight loss stage at 300~430 °C can be due to the degradation of graft chain of SSS. The last weight loss stage is considered to be the degradation of EVOH backbone. The TGA measurements can prove the successful preparation and the thermal stability of AIEM. It indicates that AIEM has good thermal stability for application in VRFB.

#### 3.2.5. Surface Morphology

**Figure 5:**
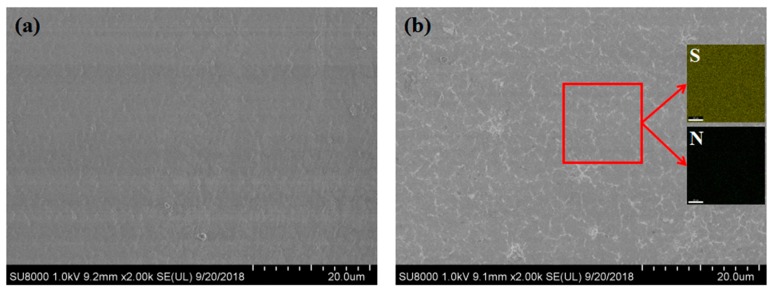
SEM image of surface morphology of EVOH film (**a**), and SEM image and EDS mapping of surface of AIEM (GY = 40.9%) (**b**).

The surface morphologies of the EVOH membrane and AIEM were represented in Figure 5. The SEM images show that the surface of the EVOH membrane is dense and smooth with no pinholes or cracks. The surface of AIEM is a bit rougher than EVOH membrane, which might be due to the micro phase separation of graft chain and EVOH. As shown in Figure 5b, the distribution of elements was studied by EDS mapping. All of elements including sulfur (from SSS) and nitrogen (from DMAEMA) are uniformly distributed in the AIEM. The SEM-EDS results demonstrate that the graft chains are evenly distributed in the AIEM.

#### 3.2.6. Elemental Analysis

The element analysis of the AIEM is shown in Table 1. It can be found that the content of nitrogen and sulfur in AIEM increase with the increasing of GY. With certain mole ratio of DMAEMA to SSS in the feed, the mole ratio of DMAEMA to SSS in the AIEM has changed little. This result demonstrates that grafting reactions occurred as expected.

#### 3.2.7. Mechanical Property

**Figure 6:**
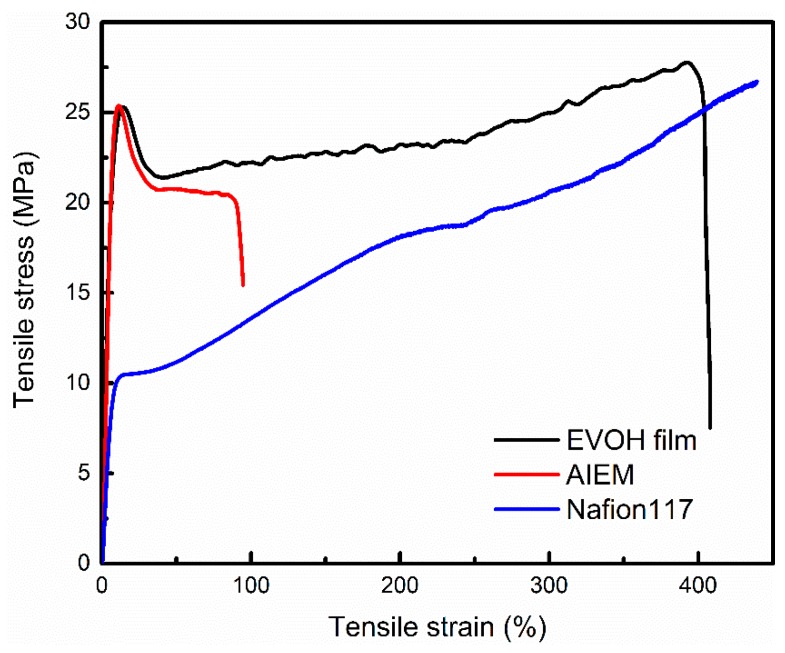
Stress-strain properties of the EVOH membrane, AIEM (GY = 40.9%) and Nafion117 membrane.

The mechanical properties of the dry EVOH membrane, AIEM (GY = 40.9%), and Nafion117 membranes were measured at room temperature, and the stress-strain curves are shown in Figure 6. The EVOH membrane and AIEM (GY = 40.9%) exhibit tensile strengths of 25.3 and 25.4 MPa, which are much higher than that of Nafion117 membrane (with a tensile strength of 10.48 MPa). Besides, EVOH membrane, AIEM, and Nafion117 membrane show elongations at break of 408%, 103% and 439%, respectively. This indicates that the AIEM has good mechanical properties and can be applied to VRFB.

#### 3.2.8. Water Uptake, Ion Exchange Capacity and Proton Conductivity

Water uptake is an important performance of the AIEM because the presence of water influences the mobility of proton. The relationship between water uptake, IEC, proton conductivity and GY is shown in Table 2. It is obvious that water uptake increases with GY, which is due to more ion exchange groups improving the hydrophilicity of AIEM with high GY. The water uptake of AIEM is higher than that of Nafion117 membrane (with water uptake of 30%). However, a high value of water uptake has an adverse effect on the mechanical properties of AIEM. Therefore, the water uptake of AIEM should be adjusted depending on actual need. The IEC and proton conductivity of the AIEM also increase with increasing ion exchange groups. When the GY is 40.9%, the IEC of AIEM was 1.05 mmol g^−1^, which is higher than Nafion117 membrane (with IEC of 0.98 mmol g^−1^). For the conductivity of AIEM, an increase in the number of ion exchange groups in a polymer has been reported to enhance the proton conductivity of the membranes [27]. Comparing with Nafion117 membrane, the conductivity of the AIEM is lower at the same IEC. This might be due to the good uniformity of Nafion117 membrane. At the IEC of AIEM is 1.05 mmol g^−1^, the conductivity of the AIEM is 41.0 mS cm^−1^ which is close to Nafion117 membrane (with conductivity of 50.1 mS cm^−1^).

#### 3.2.9. Permeability of V (IV) Ions through AIEM

**Figure 7:**
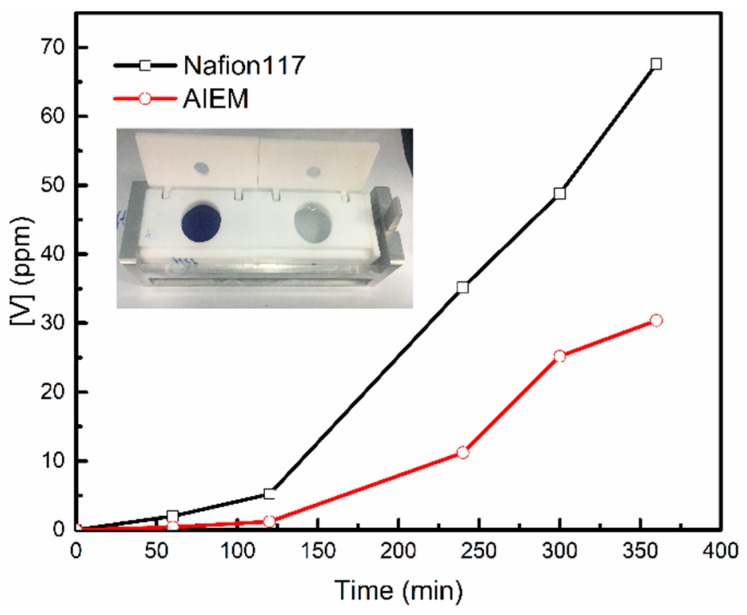
Permeability of V (IV) ions through the Nafion117 membrane and AIEM (GY = 40.9%).

The permeability of V (IV) ions is one of the most important characterizations of AIEM. The AIEM with low vanadium ion permeability could prevent the loss of energy efficiency and capacity of VRFB. The relationship between vanadium ion concentration and diffusion time of AIEM (GY = 40.9%) and Nafion117 membrane are shown in Figure 7. The permeability of V (IV) ions of membrane was calculated by Equation (6),

(6) $$V\frac{d_{c_{t}}}{d_{t}}=S\frac{P}{L}(c_{0}-c_{t})$$

where *V* is the volume of the solution in both half diffusion cell; *S* and *L* are the area and the thickness of the membrane, respectively; *P* is the permeability of V (IV) ions; *c*_0_ is the original concentration of the V (IV) ions solution and *c_t_* is concentration of the V (IV) ions in the right side at time of *t* [19].

According to Figure 7 and Equation (6), the permeability (*P*) through the AIEM (GY = 40.9%) and Nafion117 membrane were 3.98 × 10^−7^ cm^2^ min^−1^ and 12.57 × 10^−7^ cm^2^ min^−1^, respectively. The P of the V (IV) ions through the AIEM is much less than that through the Nafion117 membrane, because of the Donnan exclusion effect between V (IV) ions and –R_3_NH^+^ groups of protonated DMAEMA unit. A low permeability of vanadium ions will reduce the self-charge of VRFB. Therefore, the VRFB assembling with AIEM may show higher energy efficiency than that with Nafion117 membrane.

#### 3.2.10. Open Circuit Voltage of the VRFB

**Figure 8:**
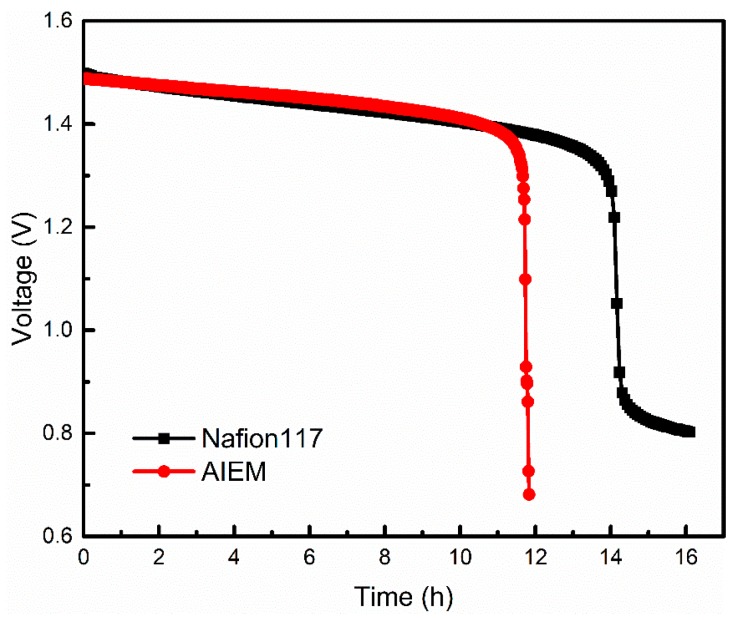
Open circuit voltage of the VRFB assembled with Nafion117 membrane and AIEM (GY = 40.9%).

The relationship between open circuit voltage and time is shown in Figure 8. It is obvious that the open circuit voltage of the VRFB assembled with Nafion117 membrane decreased after about 14h and the VRFB assembled with AIEM shows similar performance. This demonstrates that the self-discharging of VRFB with AIEM is similar to that VRFB with Nafion117 membrane. It is noteworthy that the cost of AIEM is far below Nafion117 membrane. It is expected that this work provides a simple method for preparation of AIEM with low cost and good performance for VRFB.

## 4. Discussion

In summary, a novel EVOH-based AIEM was successfully synthesized by the combination of one step radiation grafting of SSS and DMAEMA, and a casting method. The synthesis method of the AIEM was simple and cheap, which has not reported. The structure of AIEM was proved by FT-IR, TGA, SEM-EDS and elemental analysis. At the GY of 40.9%, the water uptake, IEC, tensile strength, and permeability of vanadium ions of AIEM were 96.0%, 1.05 mmol g^−1^, 25.4MPa and 3.98 × 10^−7^ cm^2^ min^−1^, respectively, which are better than that of Nafion117 membrane. Consequently, this work preliminary provided an inexpensive and efficient ion exchange membrane for the VRFB application. It is also important that this work expands the application fields of EVOH.

## Figures and Tables

**Table 1:** The element analysis of the AIEM with different GY.

DMAEMA/SSS in the Feed	Weight Percent (%)			GY (%)	DMAEMA/SSS in the AIEM
	C	N	S		
0.3:1	59.13	0.38	2.63	15.2	0.33
0.3:1	57.67	0.47	3.37	31.6	0.32
0.3:1	56.97	0.55	3.59	38.7	0.35
0.3:1	56.02	0.61	3.78	40.9	0.37

**Table 2:** The water uptake, IEC, conductivity and thickness of AIEM with different GY and Nafion117 membrane.

Sample	GY (%)	WU (%)	IEC (mmol g^−1^)	Conductivity (mS/cm)	Thickness (μm)
AIEM	15.2	34.7	0.43	3.4	118
	31.6	61.3	0.73	29.9	121
	38.7	81.2	0.94	35.2	123
	40.9	96.0	1.05	40.0	120
Nafion117	-	30.0	0.98	50.1	175

## Supplementary Materials

The following are available online at https://www.mdpi.com/2073-4360/11/5/843/s1, Figure S1: 1H NMR spectra of original EVOH and grafted EVOH powder (GY = 15.2%). Figure S2: XRD patterns of original EVOH and AIEM (GY = 40.9%).
