# Supplementary material for: Facile Preparation of EVOH-Based Amphoteric Ion Exchange Membrane Using Radiation Grafting Technique: A Preliminary Investigation on Its Application for Vanadium Redox Flow Battery
Source: Polymers (Basel). 2019 May 10;11(5):843. doi: 10.3390/polym11050843 (PMC6572321; doi:10.3390/polym11050843)
Supplement: Supplementary file 1 [file polymers-11-00843-s001.pdf]

Supporting information

Facile preparation of EVOH-based amphoteric ion exchange membrane using radiation grafting technique: A preliminary investigation on its application for vanadium redox flow battery

Kangjun Xie [1,2], Zhen Dong [1], Yicheng Wang [3], Wei Qi [1,2], Maolin Zhai [3], Long Zhao [1,*]

1 State Key Laboratory of Advanced Electromagnetic Engineering and Technology, School of Electrical and Electronic Engineering, Huazhong University of Science and Technology, 430074 Wuhan, China
2 School of Chemistry and Chemical Engineering, Huazhong University of Science and Technology, Wuhan 430074, China
3 Beijing National Laboratory for Molecular Sciences, Radiochemistry and Radiation Chemistry Key Laboratory of Fundamental Science, the Key Laboratory of Polymer Chemistry and Physics of the Ministry of Education, College of Chemistry and Molecular Engineering, Peking University, Beijing 100871, China
* Correspondence: zhaolong@hust.edu.cn (Long Zhao)

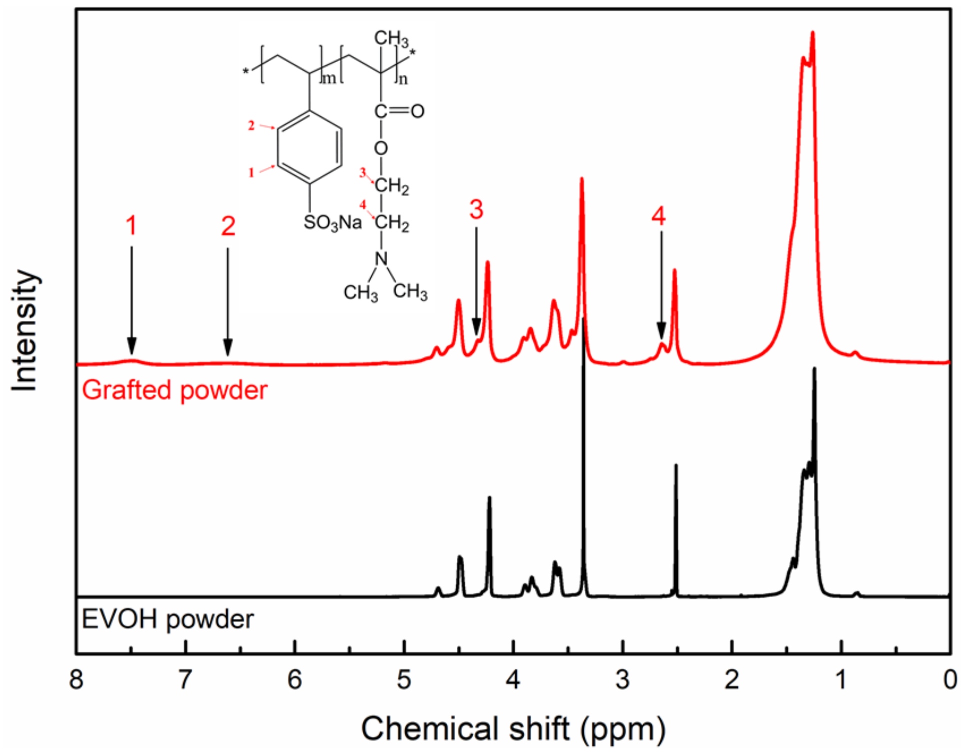

Figure. S1 ^{1}H NMR spectra of original EVOH and grafted EVOH powder (GY= 15.2%).

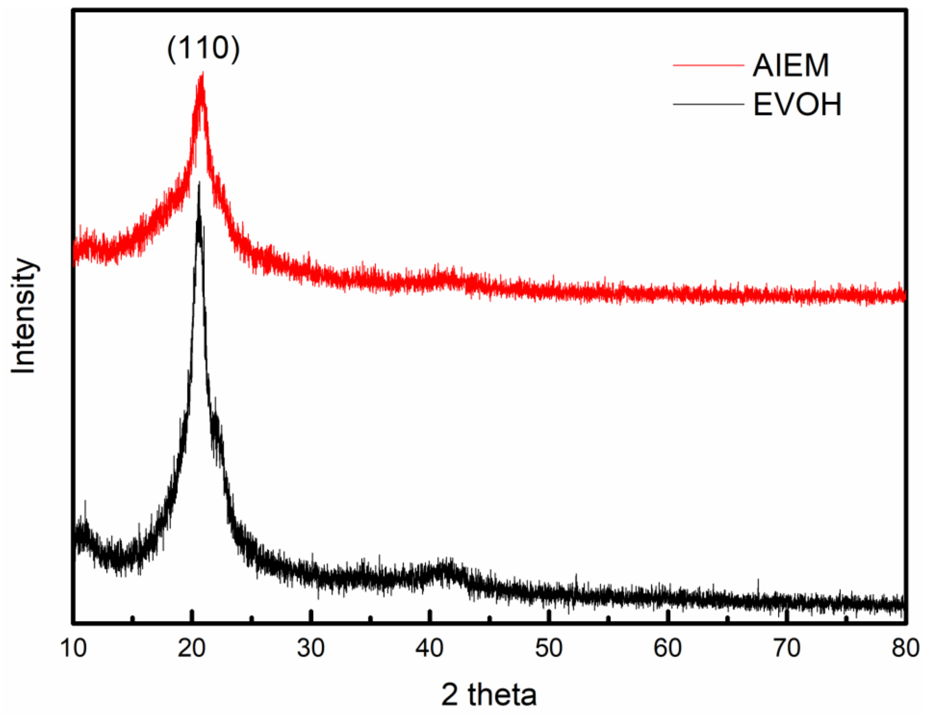

Figure. S2 XRD patterns of original EVOH and AIEM (GY= 40.9%).
